# Prognostic significance of interleukin-17A-producing colorectal tumour antigen-specific T cells

**DOI:** 10.1038/s41416-021-01283-3

**Published:** 2021-03-05

**Authors:** Amanda Thomson, Diana F. Costa Bento, Martin J. Scurr, Kathryn Smart, Michelle S. Somerville, Åsa V. Keita, Awen Gallimore, Andrew Godkin

**Affiliations:** 1 Division of Infection & Immunity, Henry Wellcome Building, Cardiff University, Cardiff, UK; 2 Division of Surgery, Orthopedics & Oncology, Department of Clinical and Experimental Medicine, Linköping University, Linköping, Sweden; 3 Department of Gastroenterology & Hepatology, University Hospital of Wales, Heath Park, Cardiff, UK

**Keywords:** Tumour immunology, Colorectal cancer

## Abstract

**Background:**

The T cell cytokine profile is a key prognostic indicator of post-surgical outcome for colorectal cancer (CRC). Whilst T_H_1 (IFN-γ^+^) cell-mediated responses generated in CRC are well documented and are associated with improved survival, antigen-specific T_H_17 (IL-17A^+^) responses have not been similarly measured.

**Methods:**

We sought to determine the cytokine profile of circulating tumour antigen-(5T4/CEA) specific T cells of 34 CRC patients to address whether antigen-specific IL-17A responses were detectable and whether these were distinct to IFN-γ responses.

**Results:**

As with IFN-γ-producing T cells, anti-5T4/CEA T_H_17 responses were detectable predominantly in early stage (TNM I/II) CRC patients. Moreover, whilst IL-17A was always produced in association with IFN-γ, this release was mainly from two distinct T cell populations rather than by ‘dual producing’ T cells. Patients mounting both tumour-specific T_H_1^+^/T_H_17^+^ responses exhibited prolonged relapse-free survival.

**Conclusions:**

Tumour antigen-specific T_H_17 responses play a beneficial role in preventing post-operative colorectal tumour recurrence.

## Background

The adaptive immune system plays a critical role in the control of colorectal cancer (CRC). Our previous work identified that the presence of pre-operative circulating T_H_1 responses specific for 5T4, an oncofoetal antigen not expressed in healthy adult tissue, has a positive association with post-operative relapse-free survival,^1^ in keeping with studies demonstrating that tumour infiltration of T_H_1-type (IFN-γ-producing) T cells associate with favourable outcome.^2^ Unexpectedly, the presence of T_H_1 responses directed towards carcinoembryonic antigen (CEA), an auto-antigen expressed at low levels in normal intestinal epithelium, associated with poor relapse-free survival in CRC patients.^1^ Whilst both CEA and 5T4 are upregulated in the majority of colorectal tumours, there is a potential that tumour-selective T cell responses impact clinical outcome differently to auto-antigen T cell responses.^3^

Since T_H_1 responses are usually associated with control of cancer progression, we postulated that CEA-specific IFN-γ-producing T cells were not the cause of the observed association with poor survival, but a surrogate marker for skewed tumour-specific T cell functionality. In particular, tumour-infiltrating T_H_17 cells have previously been associated with CRC progression.^2^ IL-17A itself has been implicated in tumour progression through STAT3 activation, promoting colonic epithelial cell transformation, tumour cell proliferation and invasion.^4^ Contrastingly, the presence of colorectal tumour-infiltrating T_H_17 cells was recently reported to correlate with improved survival,^5^ implying that IL-17A has important context and tissue-dependent roles in enacting pro- or anti-tumour immune mechanisms.^6^

This current study was designed to identify the presence of CEA-specific and 5T4-specific IL-17A- and IFN-γ-producing T cells within the blood of CRC patients and healthy controls and investigate their role on post-operative outcome and colonic epithelial barrier function.

## Methods

### Participants

Blood and colon samples were obtained from 34 patients undergoing colorectal cancer resection or investigative colonoscopy procedures at the University Hospital of Wales, Cardiff. Patient characteristics are summarised (Supplementary Table 1). Additional blood samples were obtained from age-matched healthy donors (age range 48–62; 5 male, 4 female). All participants gave written, informed consent personally prior to inclusion. The Wales Research Ethics Committee granted ethical approval for this study.

### Antigens

The CEA protein was split into seventy 20mer peptides and 5T4 split into forty-one 20mers, each overlapping by 10 amino acids and synthesised to >90% purity (GLBiochem, Shanghai, China). Peptides were dissolved in minimal DMSO and incorporated into peptide pools, used at a final concentration of 1.45 μg/ml/peptide. Phytohaemagglutinin (PHA; Sigma Aldrich) was used as a positive control.

### Peripheral blood mononuclear cell (PBMC) extraction and culture

PBMCs were extracted from whole blood as previously described.^1^ 5 × 10^5^ PBMCs were seeded per well in a 96-well plate in 100 μl R^+^ media supplemented with 5% human AB serum (Welsh Blood Service, Pontyclun, UK), 2 mM L-glutamine, 1 mM sodium pyruvate and 50 μg/ml penicillin/streptomycin (Gibco, Paisley, UK). PBMC lines were cultured for 14 days in the presence or absence of peptide pool, supplemented with 10 μl CellKine (purified T cell growth factors derived from pooled PHA-stimulated T cells; Helvetica Healthcare, Geneva, Switzerland), on day 3 and fresh media containing 40 IU/ml IL-2 on days 6 and 9.

### FluoroSpot assays

The human IFN-γ/IL-17A FluoroSpot kit (MabTech, Sweden) was used and performed according to the manufacturer’s instructions. Duplicate lines of cultured PBMCs were pooled together, washed and plated at 5 × 10^5^ cells/well with or without the corresponding peptide pool for direct comparison. FluoroSpot plates were incubated at 37 °C, 5% CO_2_ for 48 h. In some instances, blocking antibodies to MHC class-I (anti-HLA-A/B/B (W6/32); BioLegend, UK) and MHC class-II (anti-HLA-DR (L243); anti-HLA-DQ (1A3); BioLegend) were added at a final concentration of 10 μg/ml, incubated for 30 min before addition of antigen. Cytokine-producing PBMCs were enumerated using an automated FluoroSpot reader (CTL, Germany). Dual cytokine-secreting cells were visualised and enumerated by a computerised overlay. Positive IFN-γ and IL-17A responses were identified by a minimum of 10 and 5 spot-forming cells (SFC) per 1 × 10^5^ cultured PBMC, respectively, and a minimum 2-fold increase above background.

### Intestinal permeability measurements

Electrophysiological resistance and paracellular flux in human colonic biopsies was measured using an Ussing chamber, as previously described.^7^ Readouts of intestinal permeability were associated with the presence of IFN-γ and IL-17A CEA- and 5T4-specific T cell responses, with patients differentiated based on the median magnitude of response generated to each antigen.

### Statistical analysis

GraphPad Prism (v.8) was used for all statistical analyses. Dataset normality was tested using the Shapiro-Wilk test. Significance was determined using one-way ANOVA with corrections for multiple comparisons made using the Tukey test; a *P*-value < 0.05 was considered significant. For survival analyses using Kaplan–Meier curves, relapse-free survival was calculated from the date of surgery to the date of relapse, as determined by follow-up CT scan and/or endoscopy. Non-relapsing patients were censored at the time of last follow-up assessment (September 2019). Survival curves were compared using the log-rank test.

## Results

**Fig. 1:**
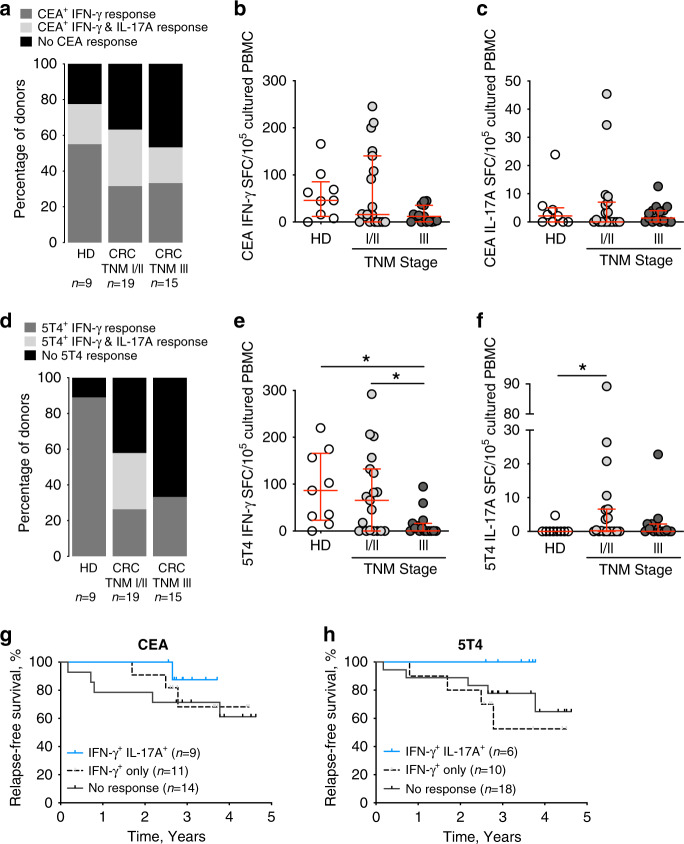
CEA- and 5T4-specific T_H_1/T_H_17 responses prevent colorectal tumour recurrence. **a** The percentage of donors mounting a CEA-specific IFN-γ response (dark grey), a dual CEA-specific IFN-γ and IL-17A response (light grey), or no positive CEA-specific response (black), as indicated by results from the IFN-γ/IL-17A FluoroSpot assay. The magnitude of CEA-specific T cell response, normalised to spot-forming cells (SFC)/10^5^ cultured PBMC, and associated with healthy donor (HD) or early-stage (TNM I/II) or late-stage (TNM III) CRC is shown for IFN-γ (**b**) and IL-17A (**c**). Error bars indicate median with interquartile range. **d** The percentage of donors mounting a 5T4-specific IFN-γ response (dark grey), a dual 5T4-specific IFN-γ and IL-17A response (light grey), or no positive 5T4-specific response (black), as indicated by results from the IFN-γ/IL-17A FluoroSpot assay. The magnitude of 5T4-specific T cell response, normalised to spot-forming cells (SFC)/10^5^ cultured PBMC, and associated with healthy donor (HD) or early-stage (TNM I/II) or late-stage (TNM III) CRC is shown for IFN-γ (**e**) and IL-17A (**f**). Error bars indicate median with interquartile range. The CEA-specific (**g**) or 5T4-specific (**h**) T_H_1/T_H_17 response measured in all CRC patients (*n* = 34) was associated with relapse-free survival.

CEA-specific and 5T4-specific IFN-γ, IL-17A and IFN-γ/IL-17A dual-secreting T cell responses from pre-operative CRC patient and age-matched healthy donor PBMC were investigated. Culturing PBMC for 14 days in the presence of a particular cytokine cocktail was necessary to expand low frequency antigen-specific T_H_17 responses to detectable levels (Supplementary Fig. 1A). Whilst T_H_17 responses were identified, IFN-γ/IL-17A dual secretion from the same cell was rarely detected; example FluoroSpot well images and results are shown for CEA and 5T4 (Supplementary Fig. 1B). CEA- and 5T4-specific T_H_1 and T_H_17 cell responses were readily identifiable amongst healthy donors and cancer patients (Supplementary Fig. 1C); these responses were predominantly MHC class II-restricted (Supplementary Fig. 1D). The proportion of cancer patients mounting detectable CEA-specific IFN-γ T cell responses diminished in comparison to healthy age-matched controls (Fig. 1a) and further declined as the tumour became more advanced (Fig. 1b). No overall change in CEA-specific IL-17A production was noted between healthy donors and CRC patients (Fig. 1c).

Similarly, 5T4-specific T_H_1 responses significantly declined as the colorectal tumour advanced (HD v III, *P* = 0.033; I/II v III, *P* = 0.031; Fig. 1d, e), validating previous findings with 5T4^1,8^ and other widely-expressed upregulated tumour antigens.^9^ 5T4-specific T_H_17 responses were rarely detectable in healthy donors, and only significantly increased in earlier stage CRC patients (*P* = 0.036, Fig. 1f).

The prognostic impact of pre-operative CEA and 5T4-specific T_H_1 and T_H_17 responses was determined by associating responses with 5-year relapse-free survival. Patients were separated based on whether they mounted both an IFN-γ and IL-17A response or not to CEA (Fig. 1g) and 5T4 (Fig. 1h); one patient that mounted an anti-5T4 IL-17A response only was included as a non-responder. Although the size of the groups did not allow statistical significance to be reached, it is clear that in both cases the most favourable immune profile pre-operatively for prolonged disease-free survival is the presence of *both* T_H_1 and T_H_17 responses (Fig. 1g, h).

From this data we conclude that release of IL-17A by CEA-specific T cells is not responsible for the poorer survival of patients with IFN-γ-producing CEA (but not 5T4)-specific T cells. Unlike 5T4, CEA is expressed at low levels in normal epithelium. A higher magnitude of IFN-γ^+^ CEA-specific T cell response, i.e. above the median of all anti-CEA responses tested in patients undergoing endoscopy (Supplementary Fig. 2A, B), was associated with decreased electrical resistance in ascending colonic epithelium (*P* = 0.037; Supplementary Fig. 2C) and increased paracellular passage (i.e. leak) of Lucifer yellow probe (*P* = 0.11; Supplementary Fig. 2D), implying that the integrity of the epithelial barrier is subtly compromised in patients with relatively high CEA T_H_1 response, a finding not replicated when separating responses based on IL-17A^+^ CEA responders, nor 5T4 responders from non-responders. This observation, specific to the right-sided ascending colonic epithelium, is worthy of further investigation.

## Discussion

Mounting pre-operative tumour antigen-specific T_H_17 and T_H_1 combined responses appears to be associated with less post-operative CRC relapse. Despite the relatively small sample size, there is a clear indication that IL-17A production by tumour antigen-specific T cells has a positive influence on anti-tumour immunity, warranting further investigation. In particular, the emergence of 5T4-specific T_H_17 response is indicative of early stage CRC and may provide diagnostic/prognostic information. This study also highlights the possibility for detrimental clinical outcomes when using IL-17/T_H_17-targeted treatments; indeed it has been suggested that clinical response to anti-PD-1 may be reliant on T_H_17 responses and treatment success is compromised by IL-17A blockade.^6,10,11^

We have previously shown an adverse effects of ex vivo measured anti-CEA T_H_1 responses on post-operative CRC patient outcome.^1^ In this previous study, we did not measure IL-17A responses, which require a period of in vitro culture and expansion. This study, albeit on smaller patient numbers, strongly suggests that T_H_17 responses are beneficial when combined with T_H_1 responses. It is possible that an IFN-γ-driven chronic, sub-clinical enteropathy which facilitates tumorigenesis is reversed or alleviated by IL-17A production.

In conclusion, tumour antigen-specific T_H_17 responses play a beneficial role in preventing colorectal tumour recurrence after surgical resection.

## Supplementary information

Supplementary Data

## Data Availability

The datasets generated during this study are available from the corresponding author on reasonable request.
