# Supplementary material for: Prognostic significance of interleukin-17A-producing colorectal tumour antigen-specific T cells
Source: Br J Cancer. 2021 Mar 5;124(9):1552–5. doi: 10.1038/s41416-021-01283-3 (PMC8076199; doi:10.1038/s41416-021-01283-3)
Supplement: Supplementary file 1 — Supplementary Data [file 41416_2021_1283_MOESM1_ESM.docx]

**Supplementary Figure 1. Establishment and characterisation of tumour antigen-specific T cell lines.**

(A) T_H_17 responses were evaluated in healthy donor PBMC stimulated with PHA or Candida albicans and cultured in the presence of indicated cytokine cocktails for 11 and 14 days. 5T4- and CEA-specific T_H_1 and T_H_17 responses were assessed after 14 days by IFN-γ/IL-17A FluoroSpot, example Fluorospot well images are shown (B) (green spots = IFN-γ, red spots = IL-17A, yellow spots = dual IFN-γ/IL-17A producing cells). (C) These responses were evaluated in 34 pre-operative CRC patients and 9 age-matched healthy controls. Cut-offs for positive / negative responses were defined based on 2 times the standard deviation of the mean of all background spot-forming cell counts per analyte (IFN-g = 10; IL-17A = 5), and a minimum 2-fold increase above background. (D) The addition of anti-HLA class II blocking antibodies but not anti-HLA class I blocking antibodies reduced anti-CEA T_H_1 and T_H_17 responses in a representative positive responder.

**Supplementary Figure 2. Associating anti-CEA/5T4 T_H_1/T_H_17 responses with readouts of intestinal permeability.**

Anti-CEA/5T4 T_H_1 and T_H_17 responses were measured in a cohort of patients undergoing endoscopy. The magnitude of IFN-γ (A) and IL-17A (B) response was separated based on the median response of all donors (red lines). Electrophysiological resistance and lucifer yellow (LY) passage across intestinal epithelial biopsy samples obtained from the ascending or descending colon were associated with anti-CEA T_H_1 response (C & D), anti-5T4 T_H_1 response (E & F), anti-CEA T_H_17 response (G & H) and anti 5T4 T_H_17 response (I & J) generated in each individual.

**Supplementary Table 1:** Characteristics of the colorectal cancer patients included in this study.

|  |  | **n = 34** |
| --- | --- | --- |
| **Male : Female** |  | 19 : 15 |
|  |  |  |
| **Age (Range)** |  | 67 (42-87) |
|  |  |  |
| **Tumour Location (%)** | Ascending | 10 (29) |
|  | Transverse | 7 (21) |
|  | Descending | 0 (0) |
|  | Sigmoid | 10 (21) |
|  | Rectum | 7 (29) |
|  |  |  |
| **TNM Stage, 5th Edition (%)** | T1 | 3 (9) |
|  | T2 | 2 (6) |
|  | T3 | 22 (64) |
|  | T4 | 7 (21) |
| (Lymph Node Spread) | N0 | 19 (56) |
|  | N1 | 11 (32) |
|  | N2 | 4 (12) |
|  |  |  |
| **Dukes’ Stage (%)** | A | 4 (12) |
|  | B | 15 (44) |
|  | C1 | 14 (41) |
|  | C2 | 1 (3) |
|  | D | 0 (0) |
|  |  |  |
